# Derailed by the COVID-19 Economy? Older Adults' Paid Work by Intersections of Age, Gender, Race-Ethnicity, and Class

**DOI:** 10.1093/geroni/igab046.1922

**Published:** 2021-12-17

**Authors:** Sarah Flood, Phyllis Moen, Joseph Pedtke

**Affiliations:** 1 University of Minnesota, Minneapolis, Minnesota, United States; 2 University of Minnesota (Life Course Center), Minneapolis, Minnesota, United States

## Abstract

This paper addresses the uneven employment effects on older Americans (Boomers and Genxers, ages 50-75) of the COVID-19 pandemic. Drawing on monthly CPS data from January through December 2020 (IPUMS) with an intersectional approach, we first chart shifts in employment and non-employment for population subgroups defined by age, gender and race/ethnicity, including explanations for not working (unemployment, retired, disabled, not in the workforce for other reasons – NILF-other). We then examine uneven transitions --monthly individual-level shifts out of and into paid work for population subgroups, considering also disparities by educational level. We find increases in proportions unemployed, especially for women in their 50s, as well as increases in the proportions reporting they are NILF-Other, especially for Asian and Hispanic women, with small increases for Asian and Hispanic men as well. There is little change in age-graded reports of being retired, regardless of gender or race/ethnicity, though there are education-level effects.

